# Nonlinear Coupled Vibration of Electrically Actuated Arch with Flexible Supports

**DOI:** 10.3390/mi10110729

**Published:** 2019-10-28

**Authors:** Ze Wang, Jianting Ren

**Affiliations:** School of Mechanics, Civil Engineering and Architecture, Northwestern Polytechnical University, Xi’an 710129, Shaanxi Province, China; wangze0127@mail.nwpu.edu.cn

**Keywords:** electrically actuated arch microbeam, flexible support, two-to-one internal resonance, nonlinear vibration

## Abstract

The nonlinear coupled vibration of an electrically actuated arch microbeam has attracted wide attention. In this paper, we studied the nonlinear dynamics of an electrically actuated arch microbeam with flexible supports. The two-to-one internal resonance between the first and second modes is considered. The multiple scales method is used to solve the governing equation. Four first-order ordinary differential equation describing the modulation of the amplitudes and phase angles were obtained. The equilibrium solution and its stability are determined. In the case of the primary resonance of the first mode, stable periodic motions and modulated motions are determined. The double-jumping phenomenon may occur. In the case of the primary resonance of the second mode, single-mode and two-mode solutions are possible. Moreover, double-jumping, hysteresis, and saturation phenomena were found. In addition, the approximate analytical results are supported by the numerical results.

## 1. Introduction

Electrically actuated microbeams are widely used in many micro-electro-mechanical systems (MEMS) devices, such as actuators, mechanical memory sensors, energy harvesters, and filters [1,2,3,4,5,6]. There is a lot of published literature on the modeling, static, and dynamic behaviors for electrically actuated microbeams [7,8,9]. To develop devices with more potential, electrically actuated arch microbeams have attracted the interest of many scientists due to their bi-stable and rich dynamic behaviors [10,11,12]. 

Internal resonance in structures is a promising and interesting nonlinear phenomenon [13,14,15,16,17,18,19,20,21,22,23,24,25,26,27]. It is capable of coupled nonlinearly vibrational modes in structures and can then exchange energy between involving modes. Much literature on internal resonance has been published. For example, Younis et al. [28] first investigated the three-to-one internal resonance of an electrically actuated microbeam. Dario et al. [29,30] explored the possibility to stabilize the oscillation frequency of nonlinear micromechanical resonators by coupling two different vibrational modes through internal resonance. Chen et al. [31] demonstrate a novel strategy to support stable oscillations in nonlinear micromechanical oscillators. Prashant et al. [32] propose a method to increase the frequency bandwidth of a microbeam array by coupling different modes. Moreover, there is a lot of literature on the internal resonance of electrically actuated arch microbeams. For example, Wang et al. [33] studied three-to-one internal resonance between the first and third modes of a MEMS arch resonator. Alfosail et al. [34,35] studied the two-to-one internal resonance between the first and third modes of a micromachined arch resonator. Hajjaj et al. [36] investigated its one-to-one internal resonance. All of this literature found that the first and third modes are coupled. Recently, Ouakad et al. [37] determined the one-to-one and three-to-one internal resonance between the first and second modes in a MEMS arch resonator, and found that although the frequency ratio of involving modes is an integer, these modes cannot be coupled and, therefore, energy exchange cannot occur.

Furthermore, in the above literature, the boundary condition of micro-structures is modeled as perfectly rigid, e.g., clamped. In fact, the clamped boundary condition is hard to achieve practically. The clamped ends are an idealization of the actual instance, which in fact has some elasticity due to fabrication imperfections [28,38]. Recent investigations have shown that flexible supports have a significant effect on the static and dynamic behavior of the structure. Ekici et al. [39] found that the frequencies with flexible boundary conditions may shift to the right or leftwith respect to the clamped boundary. Alkharabsheh et al. [40,41] found that flexible supports significantly affect the quantitative dynamics of the structures, such as changing their natural frequencies, amplitude of vibrations, snap-through, and pull-in behavior. Therefore, it is more reasonable to model the real complex boundaries as elastic supports.

Although the above research has found many complex and interesting dynamic behaviors of electrically actuated arch microbeams with elastic supports, the literature only considers single-mode dynamics. There is no investigation on the modal interaction.

Therefore, the aim of this study is to investigate the dynamics of an electrically actuated arch microbeam with elastic supports. The two-to-one internal resonance between the first and second mode is considered. The method of multiple scales is used to analyze the dynamics of the electrically actuated arch microbeam. The effect of different system parameters on the frequency response curves is also investigated. In addition, the analytical results are compared to those of the numerical method.

## 2. Problem Formulation

**Figure 1:**
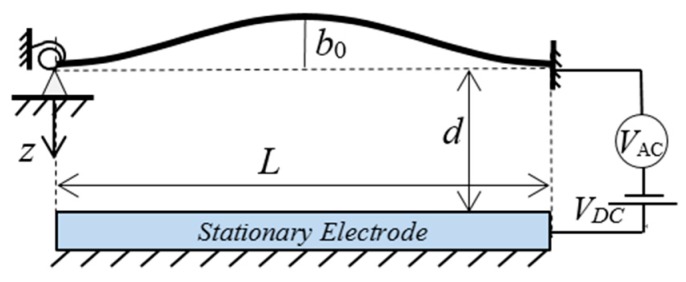
Schematic of an electrically actuated shallow arch with flexible boundary.

We model the imperfect microbeam as a shallow arch with an small initial rise, *b*_0_. The schematic of the electrically actuated shallow arch is shown in Figure 1. The length, width, and thickness of the arch are *L*, *b*, and *h*, respectively. The initial gap between the electrode is *d*. The arch is actuated by the electric load composed of DC voltage, *V*_DC_, and AC voltage, *V*_AC_. Assuming the deflection of the arch is denoted by $\hat{w}\left(\hat{x},\hat{t}\right)$ and the initial shape is denoted by ${\hat{w}}_{0}\left(\hat{x},\hat{t}\right)=0.5b_{0}\left(1-\cos2\pi \hat{x}\right)$.

In the present study, the equation of motion is obtained by assuming that:

1. The arch is shallow, i.e., $d{\hat{w}}_{0}/dx\ll 1$; hence, the parallel-plate assumption is valid;
2. As the microbeam is slender, the Euler-Bernoulli beam theory is used, neglecting the effect of shear and rotary inertia;
3. The simplest viscous damping model is adopted to model the dissipative mechanisms of the resonator;
4. As the size of the structure is small, the size effects are considered.

Based on the modified couples stress theory and Hamilton’s principle, the governing equation of the electrically actuated arch can be written as [36,42]

(1) $$\begin{array}{l} \rho A\frac{\partial^{2}\hat{w}}{\partial {\hat{t}}^{2}}+c_{d}\frac{\partial \hat{w}}{\partial \hat{t}}+\left(EI+\mu Al^{2}\right)\frac{\partial^{4}\hat{w}}{\partial {\hat{x}}^{4}}+P_{0}\left(\frac{\partial^{2}\hat{w}}{\partial {\hat{x}}^{2}}+\frac{d^{2}{\hat{w}}_{0}}{d{\hat{x}}^{2}}\right)\\ \;\;-\frac{EA}{2L}\left(\frac{\partial^{2}\hat{w}}{\partial {\hat{x}}^{2}}+\frac{d^{2}{\hat{w}}_{0}}{d{\hat{x}}^{2}}\right)\int_{0}^{L}\left[{\left(\frac{\partial \hat{w}}{\partial \hat{x}}\right)}^{2}+2\frac{\partial \hat{w}}{\partial \hat{x}}\frac{d{\hat{w}}_{0}}{d\hat{x}}\right]d\hat{x}=-\frac{\varepsilon_{r}b{\left(V_{DC}+V_{AC}\cos\hat{\Omega }t\right)}^{2}}{2{\left(d+{\hat{w}}_{0}+\hat{w}\right)}^{2}} \end{array}$$

The fixed boundary condition at the right end demands that

(2) $$\hat{w}=0,\frac{\partial \hat{w}}{\partial \hat{x}}=0\;\mathrm{at}\;\hat{x}=L$$

Because the left end is restrained by a linear torsional spring, one yields

(3) $$\hat{w}=0,\;{\hat{K}}_{L}\frac{\partial \hat{w}}{\partial \hat{x}}-EI\frac{\partial^{2}\hat{w}}{\partial {\hat{x}}^{2}}=0\;\mathrm{at}\;\hat{x}=0$$

where *ρ*, *E*, *μ*, *A*, and *I* are the materials density, the Young’s modules, the Lame’s constants, the cross-sectional area, and the moment of inertia, respectively. *l* is the material length scale parameter to characterize the size effect [18,19,43,44,45,46,47,48,49,50,51,52,53,54,55,56,57,58,59,60,61]. *ĉ* is the viscous damping coefficient. *P*_0_ is the axial load. *ε_r_* is the dielectric constant of the air. ${\hat{K}}_{L}$ is the spring stiffness.

For convenience, the equation of motion governing the transverse deflection and the boundary condition can be cast into dimensionless forms by the following parameters:

(4) $$w=\hat{w}/d,\;x=\hat{x}/L,\;T=\sqrt{\rho AL^{4}/EI},\;t=\hat{t}/T,\;\Omega =\hat{\Omega }T$$

Bt substituting Equation (4) into Equations (1)–(3), we obtain:

(5) $$\begin{array}{c} \ddot{w}+2c\dot{w}+k_{1}w^{iv}+\left(\frac{\partial^{2}w}{\partial x^{2}}+\frac{d^{2}w_{0}}{dx^{2}}\right)\left(P-\alpha_{1}\int_{0}^{L}\left[{\left(\frac{\partial w}{\partial x}\right)}^{2}+2\frac{\partial w}{\partial x}\frac{dw_{0}}{dx}\right]d\hat{x}\right)\\ =-\frac{\alpha_{2}{\left(V_{DC}+V_{AC}\cos\left(\Omega t\right)\right)}^{2}}{{\left(1+w_{0}+w\right)}^{2}} \end{array}$$

(6) $$w\left(0,t\right)=w^{\prime }\left(0,t\right)=0\;w\left(1,t\right)=0\;w^{\prime }\left(0,t\right)-K_{L}w^{\prime \prime }\left(0,t\right)=0$$

where the overdot indicates the derivative with respect to *t*, the prime indicates the derivative to *x*, and

(7) $$\begin{array}{l} k_{1}=\frac{EI+\mu Al^{2}}{EI},\;\alpha_{1}=6{\left(\frac{d}{h}\right)}^{2},\;\alpha_{2}=\frac{\varepsilon_{r}bL^{4}}{2EId^{3}},\;2c=\frac{c_{d}L^{4}}{EIT},\;P=\frac{\hat{P}L^{2}}{EI}\\ w_{0}=-\frac{b_{0}}{2d}\left[1-\cos\left(2\pi x\right)\right],\;K_{L}=\frac{EI}{{\hat{K}}_{L}L} \end{array}$$

The arch deflection under an electric load consists of a static component due to the DC voltage and a dynamic component due to the AC voltage. The static component can be obtained from Equations (5) and (6) by dropping time-dependent terms and denoting static deformation by *ψ*(*x*). The result is

(8) $$k_{1}\psi^{iv}+\left(\frac{\partial^{2}\psi }{\partial x^{2}}+\frac{d^{2}w_{0}}{dx^{2}}\right)\left(P-\alpha_{1}\int_{0}^{L}\left[{\left(\frac{\partial \psi }{\partial x}\right)}^{2}+2\frac{\partial \psi }{\partial x}\frac{dw_{0}}{dx}\right]d\hat{x}\right)=-\frac{\alpha_{2}V_{\mathrm{DC}}^{2}}{{\left(1+w_{0}+\psi \right)}^{2}}$$

(9) $$\psi \left(0,t\right)=\psi^{\prime }\left(0,t\right)=0\psi^{\prime }\left(1,t\right)=0\psi^{\prime }\left(0,t\right)-K_{L}\psi^{\prime \prime }\left(0,t\right)=0$$

We assume that *w*_s_(*x*) denotes a static equilibrium position in coordinates. Then

(10) $$w_{s}\left(x\right)=\psi \left(x\right)+w_{0}\left(x\right)$$

Substituting Equation (10) into Equations (8) and (9) and then multiplying the result by (1+*w_s_*)^2^ yields

(11) $$k_{1}{\left(1+w_{s}\right)}^{2}w_{s}^{iv}+{\left(1+w_{s}\right)}^{2}\left[P-\alpha_{1}\int_{0}^{1}\left({w^{\prime }}_{s}^{2}-{w^{\prime }}_{0}^{2}\right)dx\right]{w^{\prime \prime }}_{s}+\alpha_{2}V_{DC}^{2}=0$$

The boundary conditions are:

(12) $$w_{s}\left(0,t\right)={w^{\prime }}_{s}\left(0,t\right)=0\;w_{s}\left(1,t\right)=0\;{w^{\prime }}_{s}\left(0,t\right)-K_{L}{w^{\prime \prime }}_{s}\left(0,t\right)=0$$

To obtain the governing equation of the arch vibration, we assume that a dynamical disturbance *υ*(*x*, *t*) takes place around the static equilibrium position. Then,

(13) $$w\left(x,t\right)=w_{s}\left(x\right)+\upsilon \left(x,t\right)$$

Substituting Equation (13) into Equations (5) and (6), and using Equations (11) and (12) to eliminate the terms representing the equilibrium position. To third-order in *υ*, the result is:

(14) $$\begin{array}{ll} \ddot{\upsilon }+2c\dot{\upsilon }+ & k_{1}\upsilon^{iv}+\lambda^{2}\upsilon^{\prime \prime }-2\alpha_{1}{w^{\prime \prime }}_{s}\int_{0}^{1}{w^{\prime }}_{s}\upsilon^{\prime }dx-\alpha_{1}{w^{\prime \prime }}_{s}\int_{0}^{1}{\upsilon^{\prime }}^{2}dx\\ & -2\alpha_{1}\upsilon^{\prime \prime }\int_{0}^{1}{w^{\prime }}_{s}\upsilon^{\prime }dx-\alpha_{1}\upsilon^{\prime \prime }\int_{0}^{1}{\upsilon^{\prime }}^{2}dx-\frac{2\alpha_{2}V_{DC}^{2}}{{\left(1+w_{s}\right)}^{3}}\upsilon \\ & -\frac{3\alpha_{2}V_{DC}^{2}}{{\left(1+w_{s}\right)}^{4}}\upsilon^{2}-\frac{4\alpha_{2}V_{DC}^{2}}{{\left(1+w_{s}\right)}^{5}}\upsilon^{3}=-2\alpha_{2}V_{DC}V_{AC}\cos\left(\Omega t\right) \end{array}$$

(15) $$\upsilon \left(0,t\right)=\upsilon^{\prime }\left(0,t\right)=0\;\upsilon \left(1,t\right)=0\;\upsilon^{\prime }\left(0,t\right)-K_{L}\upsilon^{\prime \prime }\left(0,t\right)=0$$

where $\lambda^{2}=P-\alpha_{1}\int_{0}^{1}\left({w^{\prime }}_{s}^{2}-{w^{\prime }}_{0}^{2}\right)dx$.

## 3. The Method of Multiple Scales

In this section, we use the multiple scales method to solve Equations (14) and (15). In the presence of two-to-one internal resonance, the primary resonance of the first and second mode is considered. To this end, we introduced the two independent time scales:

(16) $$T_{0}=t,\;T_{1}=\varepsilon t$$

where *ε* is a small dimensionless parameter. It follows that the derivatives with respect to *t* can be expressed as expansion in terms of the partial derivatives with respect to the *T_n_*

(17) $$\begin{array}{l} \frac{\partial }{\partial t}=\frac{\partial }{\partial T_{0}}+\varepsilon \frac{\partial }{\partial T_{1}}+\varepsilon^{2}\frac{\partial }{\partial T_{2}}=D_{0}+\varepsilon D_{1}+\varepsilon^{2}D_{2}\\ \frac{\partial^{2}}{\partial t^{2}}=D_{0}^{2}+2\varepsilon D_{0}D_{1}+\varepsilon^{2}\left(D_{1}^{2}+2D_{0}D_{2}\right)+\ldots \end{array}$$

The solution of Equation (14) can be represented by an expansion having the form:

(18) $$\upsilon \left(x,t;\varepsilon \right)=\varepsilon \upsilon_{1}\left(x,T_{0},T_{1},T_{2}\right)+\varepsilon^{2}\upsilon_{2}\left(x,T_{0},T_{1},T_{2}\right)$$

In order that the damping coefficients and the external excitation term appear in the same perturbation equation as the quadratic nonlinear terms, we let *c* = *εc*, *V*_AC_ = *ε*^2^*V*_AC_. Substituting Equations (16)–(18) into the governing equation, equating coefficients of like power of *ε*, we obtain:

(19) $$D_{0}^{2}\upsilon_{1}+k_{1}\upsilon_{1}^{iv}+\lambda^{2}{\upsilon^{\prime \prime }}_{1}-2\alpha_{1}{w^{\prime \prime }}_{s}\int_{0}^{1}{w^{\prime }}_{s}{\upsilon^{\prime }}_{1}dx-\frac{2\alpha_{2}V_{DC}^{2}}{{\left(1+w_{s}\right)}^{3}}\upsilon_{1}=0$$

(20) $$\begin{array}{ll} D_{0}^{2}\upsilon_{2}+ & k_{1}\upsilon_{2}^{iv}+\lambda^{2}{\upsilon^{\prime \prime }}_{2}-2\alpha_{1}{w^{\prime \prime }}_{s}\int_{0}^{1}{w^{\prime }}_{s}{\upsilon^{\prime }}_{2}dx-\frac{2\alpha_{2}V_{DC}^{2}}{{\left(1+w_{s}\right)}^{3}}\upsilon_{2}\\ & \;=\alpha_{1}{w^{\prime \prime }}_{s}\int_{0}^{1}{\upsilon^{\prime }}_{1}^{2}dx+2\alpha_{1}{\upsilon^{\prime \prime }}_{1}\int_{0}^{1}{w^{\prime }}_{s}{\upsilon^{\prime }}_{1}dx-\frac{3\alpha_{2}V_{DC}^{2}}{{\left(1+w_{s}\right)}^{4}}\upsilon_{1}^{2}-2\alpha_{2}V_{DC}V_{AC}\cos\left(\Omega t\right) \end{array}$$

where

(21) $$\upsilon \left(0,t\right)=\upsilon \left(1,t\right)=0\;\upsilon^{\prime }\left(0,t\right)-K_{L}\upsilon^{\prime \prime }\left(0,t\right)=0\;\upsilon^{\prime }\left(1,t\right)=0$$

Equations (19) is the linear eigenvalue problem of the arch. The solution of Equation (19) can be expressed as:

(22) $$\upsilon_{1}\left(x,T_{0},T_{2}\right)=\sum_{m=1}^{\infty }A_{m}\left(T_{2}\right)\phi_{m}\left(x\right)e^{i\omega_{m}T_{0}}+cc$$

where *A_m_*(*T*_2_) denotes the complex function to be determined, *ω_m_* is the *m*th natural frequency of the system, *ϕ_m_*(*x*) is the mode function, and *cc* indicates the complex conjugate of the preceding terms.

**Figure 2:**
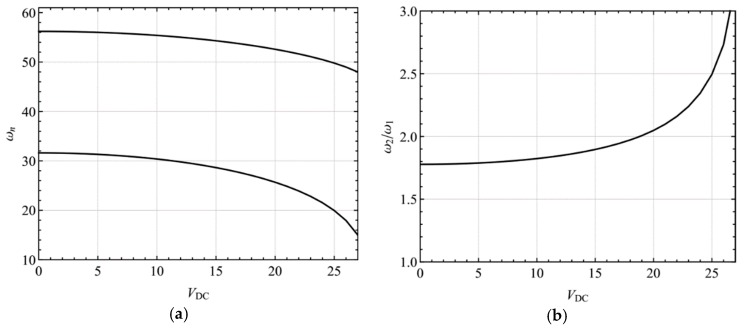
(**a**) Natural frequency variation against *V*_DC_; (**b**) variation of the frequency ratio between the second and first natural frequencies with *V*_DC_.

The linear eigenvalue problem has been studied by many researchers [62]. As a case study, we choose dimensionless parameters as *k*_1_ = 1.03, *K_L_* = 0.10, *P* = 5, *α*_1_ = 75.62, and *α*_2_ = 0.11. Here, Figure 2a shows the variation of the first and second natural frequencies with the DC voltage; Figure 2b shows variations of the frequency ratio with the DC voltage. It is found that when the DC voltage is fixed at *V*_DC_ = 18.82, the frequency ratio between the second and first natural frequencies is 2. Hence, a two-to-one internal resonance between the first and second modes may be activated when *V*_DC_ is near 18.82. Here, we assumed that neither of these two modes is involved in an internal resonance with the other modes. Due to the presence of damping and internal resonance, only the first and second modes will contribute to the long-time dynamic response. As a result, the solution of Equation (19) can be written in the form:

(23) $$\upsilon_{1}\left(x,T_{0},T_{2}\right)=A_{1}\left(T_{2}\right)\phi_{1}\left(x\right)e^{i\omega_{1}T_{0}}+A_{2}\left(T_{2}\right)\phi_{2}\left(x\right)e^{i\omega_{2}T_{0}}+cc$$

where *A*_1_ and *A*_2_ are unknown complex functions. *ϕ*_1_ and *ϕ*_2_ are the first and second modal shapes, respectively. *cc* is the conjugate of the previous terms.

Substituting Equation (23) into Equation (20) yields

(24) $$\begin{array}{ll} L\left(\upsilon_{2}\right) & =\left(-2i\omega_{1}D_{1}A_{1}\phi_{1}-2ic\omega_{1}A_{1}\phi_{1}\right)e^{i\omega_{1}T_{0}}+\left(-2i\omega_{2}D_{1}A_{2}\phi_{2}-2ic\omega_{2}A_{2}\phi_{2}\right)e^{i\omega_{2}T_{0}}\\ & +h_{11}A_{1}^{2}e^{i\omega_{n}T_{0}}e^{-i\sigma_{1}T_{0}}+H_{12}A_{2}{\bar{A}}_{1}e^{i\omega_{1}T_{0}}e^{i\sigma_{1}T_{0}}+h_{22}A_{2}^{2}e^{i2\omega_{2}T_{0}}\\ & +2h_{11}A_{1}{\bar{A}}_{1}+2h_{22}A_{2}{\bar{A}}_{2}+H_{12}A_{1}A_{2}e^{i\left(\omega_{1}+\omega_{2}\right)T_{0}}-\frac{\alpha_{2}V_{DC}V_{AC}}{{\left(1+w_{s}\right)}^{2}}e^{i\Omega T_{0}}+\mathrm{cc} \end{array}$$

where *cc* is the complex conjugate of the preceding terms and $h_{1i}=\alpha_{1}\left({w^{\prime \prime }}_{s}\int_{0}^{1}{\phi^{\prime }}_{i}^{2}dx+2{\phi^{\prime \prime }}_{i}\int_{0}^{1}{w^{\prime }}_{s}{\phi^{\prime }}_{i}dx\right)-\frac{3\alpha_{2}V_{DC}^{2}}{{\left(1+w_{s}\right)}^{4}}\phi_{i}^{2}$ *i* = 1, 2

$$H_{12}=2\alpha_{1}\left({w^{\prime \prime }}_{s}\int_{0}^{1}{\phi^{\prime }}_{1}{\phi^{\prime }}_{2}dx+{\phi^{\prime \prime }}_{1}\int_{0}^{1}{w^{\prime }}_{s}{\phi^{\prime }}_{2}dx+{\phi^{\prime \prime }}_{2}\int_{0}^{1}{w^{\prime }}_{s}{\phi^{\prime }}_{1}dx\right)-\frac{6\alpha_{2}V_{DC}^{2}}{{\left(1+w_{s}\right)}^{4}}\phi_{1}\phi_{2}$$

### 3.1. Primary Resonance of the First Mode

To describe the nearness of the *ω*_2_ to 2*ω*_1_ and the excitation frequency Ω to *ω*_1_, we let

(25) $$\omega_{2}=2\omega_{1}+\varepsilon \sigma_{1}\;\;\Omega =\omega_{1}+\varepsilon \sigma_{2}$$

where *σ*_1_ and *σ*_2_ are the detuning parameters. Due to the homogeneous part of Equation (20) which has a nontrivial solution, Equation (20) has a nontrivial solution only if the solvability conditions are satisfied. Because this problem is self-adjoint, it can be demanded that the right hand of Equation (20) is orthogonal to *ϕ*_1_(*x*)exp(-*iω*_1_*T*_0_) and *ϕ*_2_(*x*)exp(-*iω*_2_*T*_0_). Performing these manipulations, we were able to obtain complex-valued modulation equations

(26) $$2iD_{1}A_{1}=-2icA_{1}+4R_{1}A_{2}{\bar{A}}_{1}e^{i\sigma_{1}T_{0}}-F_{1}e^{i\sigma_{2}T_{1}}$$

(27) $$2iD_{1}A_{2}=-2icA_{2}+4R_{2}A_{1}^{2}e^{-i\sigma_{1}T_{0}}$$

where

$$R_{1}=\frac{1}{4\omega_{1}}\int_{0}^{1}H_{12}\phi_{1}dx,\;R_{2}=\frac{1}{4\omega_{2}}\int_{0}^{1}h_{11}\phi_{2}dx,\;F_{1}=\frac{\alpha_{2}V_{DC}V_{AC}}{4\omega_{1}}\int_{0}^{1}\frac{\phi_{1}}{{\left(1+w_{s}\right)}^{2}}dx$$

Next, introducing the polar transformation

(28) $$A_{1}=\frac{1}{2}a_{1}e^{i\beta_{1}\left(T_{1}\right)}A_{2}=\frac{1}{2}a_{2}e^{i\beta_{2}\left(T_{1}\right)}$$

and substituting them into Equations (26) and (27), and separating real and imaginary parts, we obtain the following modulation equation:

(29) $${\dot{a}}_{1}=-ca_{1}+R_{1}a_{1}a_{2}\sin\gamma_{1}-F_{1}\sin\gamma_{2}$$

(30) $${\dot{a}}_{2}=-ca_{2}-R_{2}a_{1}^{2}\sin\gamma_{1}$$

(31) $${\dot{\beta }}_{1}a_{1}=-R_{1}a_{1}a_{2}\cos\gamma_{1}+F_{1}\cos\gamma_{2}$$

(32) $${\dot{\beta }}_{2}a_{2}=-R_{2}a_{1}^{2}\cos\gamma_{1}$$

where

(33) $$\gamma_{1}=\beta_{2}-2\beta_{1}+\sigma_{1}T_{1}\;\;\gamma_{2}=\sigma_{2}T_{1}-\beta_{1}$$

Periodic solutions of the system correspond to the equilibrium points of Equations (29)–(32), which in turn correspond to $a_{1}^{’}$ = $a_{2}^{’}$ = $\gamma_{1}^{’}$ = $\gamma_{2}^{’}$ = 0. Therefore, one can get the frequency-amplitude relationship in the first mode

(34) $$a_{1}^{6}+\Delta_{1}a_{1}^{4}+\Delta_{2}a_{1}^{2}+\Delta_{3}F_{1}^{2}=0$$

(35) $$a_{2}=\frac{\left|R_{2}\right|a_{1}^{2}}{{\left(2\sigma_{2}-\sigma_{1}\right)}^{2}+c^{2}}$$

where

$$\Delta_{1}=\frac{2c^{2}-2\sigma_{2}\left(2\sigma_{2}-\sigma_{1}\right)}{R_{1}R_{2}},\;\Delta_{2}=\frac{\left(\sigma_{2}^{2}+c^{2}\right)\left[{\left(2\sigma_{2}-\sigma_{1}\right)}^{2}+c^{2}\right]}{R_{1}^{2}R_{2}^{2}},\;\Delta_{3}=-\frac{{\left(2\sigma_{2}-\sigma_{1}\right)}^{2}+c^{2}}{R_{1}^{2}R_{2}^{2}}$$

To examine the stability of the response, we express the modulation equations as the Cartesian form. To this end, we introduce:

(36) $$A_{1}=\frac{1}{2}\left(p_{1}-iq_{1}\right)\;\;A_{2}=\frac{1}{2}\left(p_{2}-iq_{2}\right)$$

Substituting Equations (36) into Equations (26) and (27) yields:

(37) $${\dot{p}}_{1}=-cp_{1}-v_{1}q_{1}+R_{1}\left(p_{2}q_{1}-p_{1}q_{2}\right)$$

(38) $${\dot{q}}_{1}=-cq_{1}+v_{1}p_{1}+R_{1}\left(p_{1}p_{2}+q_{1}q_{2}\right)+F_{1}$$

(39) $${\dot{p}}_{2}=-cp_{2}-v_{2}q_{2}-2R_{2}p_{1}q_{1}$$

(40) $${\dot{q}}_{2}=-cq_{2}+v_{2}p_{2}+R_{2}\left(p_{1}^{2}-q_{1}^{2}\right)$$

where *v*_1_ = 2σ_1_ - σ_2_, *v*_2_ = σ_2_. The eigenvalues *η_m_* of the Jacobian matrix are determined from:

(41) $$\left|\begin{array}{cccc} \eta +c+R_{1}q_{2} & v_{1}-R_{1}p_{2} & -R_{1}q_{1} & R_{1}p_{1}\\ -v_{1}-R_{1}p_{2} & \eta +c-R_{1}q_{2} & -R_{1}p_{1} & -R_{1}q_{1}\\ 2R_{2}q_{1} & 2R_{2}p_{1} & \eta +c & v_{2}\\ -2R_{2}p_{1} & 2R_{2}q_{1} & -v_{2} & \eta +c \end{array}\right|=0$$

Equation (41) can be written as the closed-form:

(42) $$\begin{array}{l} \eta^{4}+2c^{2}\eta^{3}+\left[6c^{2}+v_{1}^{2}+v_{2}^{2}-R_{1}^{2}a_{2}^{2}+4R_{1}R_{2}a_{1}^{2}\right]\eta^{2}\\ +\left[4c^{3}+2c\left(v_{1}^{2}+v_{2}^{2}-R_{1}^{2}a_{2}^{2}\right)+8cR_{1}R_{2}a_{1}^{2}\right]\eta +\\ c^{4}+c^{2}\left(v_{1}^{2}+v_{2}^{2}\right)+v_{1}^{2}v_{2}^{2}-\left(c^{2}+v_{2}^{2}\right)R_{1}^{2}a_{2}^{2}+4\left(c^{2}-v_{1}v_{2}\right)R_{1}R_{2}a_{1}^{2}+4R_{1}^{2}R_{2}^{2}a_{1}^{4}=0 \end{array}$$

Solving Equation (38), and by then examining the sign of the eigenvalues, the stability of the equilibrium solution can be found.

### 3.2. Primary Resonance of the Second Mode

To describe the nearness of the *ω*_2_ to 2*ω*_1_ and the excitation frequency Ω to *ω*_1_, we let

(43) $$\omega_{2}=2\omega_{1}+\varepsilon \sigma_{1}\;\;\Omega =\omega_{2}+\varepsilon \sigma_{2}$$

Performing the similar processes in Section 3.1, we were able to obtain complex-valued modulation equations

(44) $$2iD_{1}A_{1}=-2icA_{1}+4R_{1}A_{2}{\bar{A}}_{1}e^{i\sigma_{1}T_{0}}$$

(45) $$2iD_{1}A_{2}=-2icA_{2}+4R_{2}A_{1}^{2}e^{-i\sigma_{1}T_{0}}-F_{2}e^{i\sigma_{2}T_{1}}$$

where $F_{2}=\frac{\alpha_{2}V_{DC}V_{AC}}{4\omega_{2}}\int_{0}^{1}\frac{\phi_{2}}{{\left(1+w_{s}\right)}^{2}}dx$, Substituting Equation (28) into Equations (44) and (45) yields:

(46) $${\dot{a}}_{1}=-ca_{1}+R_{1}a_{1}a_{2}\sin\gamma_{1}$$

(47) $${\dot{a}}_{2}=-ca_{2}-R_{2}a_{1}^{2}\sin\gamma_{1}-F_{2}\sin\gamma_{2}$$

(48) $${\dot{\beta }}_{1}a_{1}=-R_{1}a_{1}a_{2}\cos\gamma_{1}$$

(49) $${\dot{\beta }}_{2}a_{2}=-R_{2}a_{1}^{2}\cos\gamma_{1}+F_{2}\cos\gamma_{2}$$

where

(50) $$\gamma_{1}=\beta_{2}-2\beta_{1}+\sigma_{1}T_{1}\;\gamma_{2}=\sigma_{2}T_{1}-\beta_{2}$$

The equilibrium points of Equations (46)–(49) correspond to the steady-state response. There are two possible types of solutions. The first is the single-mode solution (*a*_1_ = 0, *a*_2_ ≠ 0) that can be expressed as:

(51) $$a_{1}=0,\;\;a_{2}=\frac{F_{2}}{\sqrt{c^{2}+\sigma_{2}^{2}}}$$

which is essentially the linear solution in the second mode. 

The second is the coupled two-mode solution (*a*_1_ ≠ 0, *a*_2_ ≠ 0) that can be expressed as:

(52) $$a_{1}^{4}+\frac{2c^{2}-\left(\sigma_{1}+\sigma_{2}\right)\sigma_{2}}{R_{1}R_{2}}a_{1}^{2}+\frac{\left(c^{2}+\sigma_{2}^{2}\right)\left[4c^{2}+{\left(\sigma_{1}+\sigma_{2}\right)}^{2}\right]-4R_{1}^{2}F_{2}^{2}}{4R_{1}^{2}R_{2}^{2}}=0$$

(53) $$a_{2}^{2}=\frac{1}{R_{1}^{2}}\left[c^{2}+\frac{1}{4}{\left(\sigma_{1}+\sigma_{2}\right)}^{2}\right]$$

To determine the stability of the single-mode solution, substituting *a*_1_ = 0 into Equation (42) obtains:

(54) $$\left(\eta^{2}+2c\eta +c^{2}+v_{1}^{2}-R_{1}^{2}a_{2}^{2}\right)\left(\eta^{2}+2c\eta +c^{2}+v_{2}^{2}\right)=0$$

According to the Routh-Hurwitz stability criterion, all eigenvalues are with negative real parts and then the single-mode is stable if:

(55) $$R_{1}^{2}a_{2}^{2}<c^{2}+v_{2}^{2}$$

The stability of the coupled two-mode solution can be determined by substituting the equilibrium solution (*a*_1_, *a*_2_) into Equation (42) and then examining the sign of the eigenvalues. 

## 4. Numerical Results

In this section, the numerical example of the electrically actuated shallow arch will be presented. The present study shows the results of the primary resonance of the first mode (i.e., Ω = *ω*_1_) and the second mode (i.e., Ω = *ω*_2_) in the presence of 2:1 internal resonance. In all figures, solid lines denote stable equilibrium solutions, the blue dashed lines denote unstable solutions (saddle-node), and the red solid lines denote modulated solutions (focus). 

### 4.1. Primary Resonance of the First Mode

In the case of the primary resonance of the first mode, dimensionless parameters of the system are chosen as *k*_1_ = 1.03, *P* = 5, *c* = 0.01, *K_L_* = 0.10, *V*_DC_ = 18.82, *V*_AC_ = 0.05, *α*_1_ = 75.62, and *α*_2_ = 0.11 unless other values are set. The internal resonance condition *ω*_2_: *ω*_1_ = 2 is satisfied.

**Figure 3:**
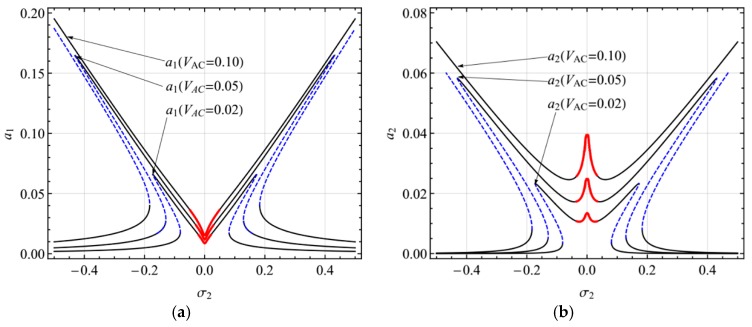
Frequency-response curves of primary resonance of the first mode for *V*_AC_ = 0.02, 0.05, 0.10: (**a**) the first mode, (**b**) the second mode.

Figure 3 shows the frequency-response curves of the primary resonance of the first mode for different AC voltage (*V*_AC_ = 0.02, 0.05, 0.10). It is seen from the figure that there are two peaks bending to the opposite directions. The double-jumping phenomena can occur via increasing or decreasing the excitation frequency. Hopf bifurcation occurs near σ_1_ = 0 and the amplitude- and phase-modulated motion may take place. Moreover, increasing the excitation, both the steady-state response amplitude and the range of resonance frequency increases.

**Figure 4:**
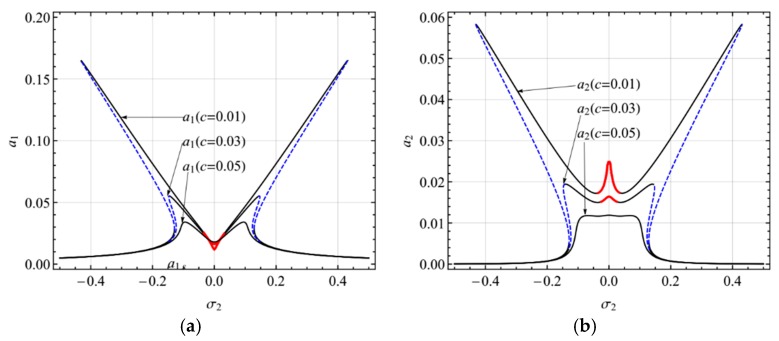
Frequency-response curves of primary resonance of the first mode for *c* = 0.01, 0.03, 0.05; (**a**) the first mode, (**b**) the second mode.

Figure 4 shows the frequency-response curves of the primary resonance of the first mode for different damping coefficients (*c* = 0.01, 0.03, 0.05). It is seen from the figure that the double-jumping phenomena can occur only for small damping coefficients. With the growth of the damping coefficients, double-jumping reduces to two peaks without jumping and the steady-state response close to *σ*_1_ = 0 becomes the stable periodic solution. Moreover, as the damping coefficients increase, the response amplitude decreases.

**Figure 5:**
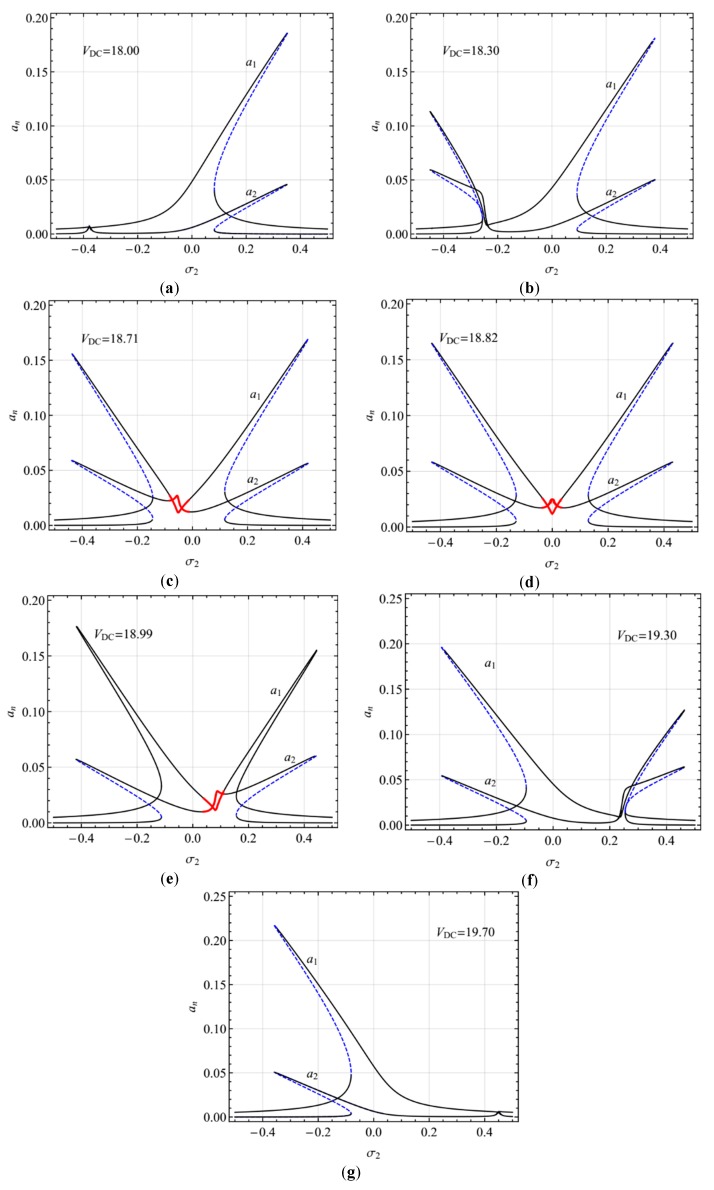
Frequency-response curves of primary resonance of the first mode for different DC voltage (**a**) *V*_DC_ = 18.00 (σ_1_ = -0.75), (**b**) *V*_DC_ = 18.30 (σ_1_ = -0.49), (**c**) *V*_DC_ = 18.71 (σ_1_ = -0.10), (**d**) *V*_DC_ = 18.82 (σ_1_ ≈ 0), (**e**) *V*_DC_ = 18.99 (σ_1_ = 0.17), (**f**) *V*_DC_ = 19.30 (σ_1_ = 0.48), (**g**) *V*_DC_ = 19.70 (σ_1_ = 0.90).

Figure 5 depicts the frequency-response curves of the primary resonance of the first mode for different DC voltages. For sufficiently small DC voltages, *V*_DC_, *σ*_1_ is small enough and the internal resonance condition cannot be meet such that only single-jumping with hardening-spring type occurs (Figure 5a). With increases in *V*_DC_, such that *σ*_1_ becomes near zero but still remains negative, an additional jumping with softening-spring type emerges to form double-jumping (Figure 5b). Further increasing *V*_DC_, the modulated motion occurs (Figure 5c). When *σ*_1_ is almost zero, double-jumping is near-symmetrical (Figure 5d). Increasing *V*_DC_ so that *σ*_1_ becomes positive and much larger, the jumping with hardening-spring type becomes much smaller (Figure 5e,f) and the modulated motion disappears (Figure 5f). Increasing *V*_DC_, *σ*_1_ becomes larger enough so that the internal resonance condition cannot be meet again, and jumping with the hardening-spring type disappears, and hence double-jumping reduces single-jumping with the softening-spring type (Figure 5g).

**Figure 6:**
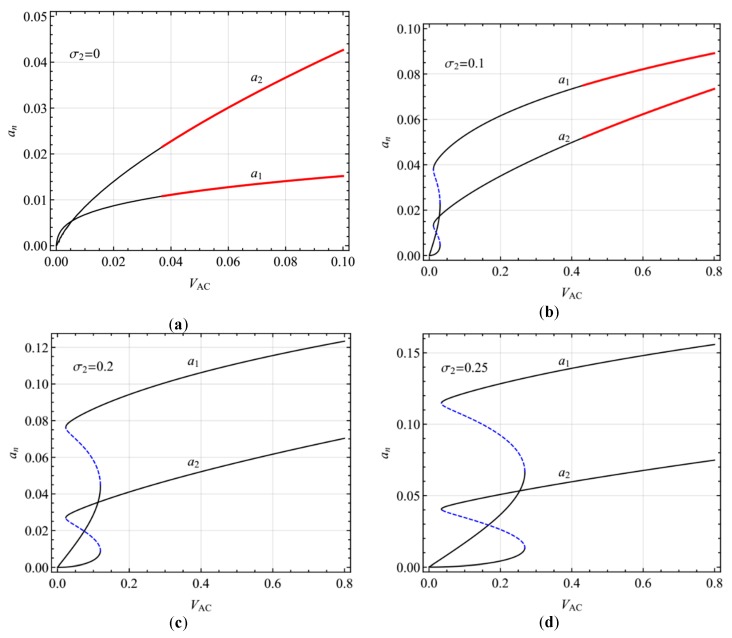
Response amplitude varying with exciting amplitude in the primary resonance of the first mode for different exciting frequencies: (**a**) σ_2_ = 0; (**b**) σ_2_ = 0.05; (**c**) σ_2_ = 0.10; (**d**) σ_2_ = 0.15.

Figure 6 shows the variation of the steady-state response amplitude with AC voltage in the presence of two-to-one internal resonances for different AC voltage frequency. At the exact primary resonance (σ_2_ = 0), there is only one steady-state response, which is stable for small AC voltages and modulated for large AC voltages (Figure 6a). When the AC voltage frequency is near the primary resonance (Figure 6b), there are two stable responses and one unstable response for small AC voltage amplitudes and the modulated response for large AC voltage amplitudes. By further increasing the AC voltage frequency, the modulated response occurs at a larger AC voltage. The multivaluedness leads to the hysteresis phenomenon (Figure 5b–d), which occurs at a larger AC voltage with increasing growth of the AC voltage frequency. 

### 4.2. Primary Resonance of the Second Mode

In the cases of the primary resonance of the second mode, dimensionless parameters of the system are chosen as *k*_1_ = 1.03, *c* = 0.01, *K_L_* = 0.10, *V*_DC_ = 18.82, *V*_AC_ = 0.05, *α*_1_ = 75.62, and *α*_2_ = 0.11 unless other values are set. The internal resonance condition *ω*_2_:*ω*_1_ = 2 is satisfied. 

**Figure 7:**
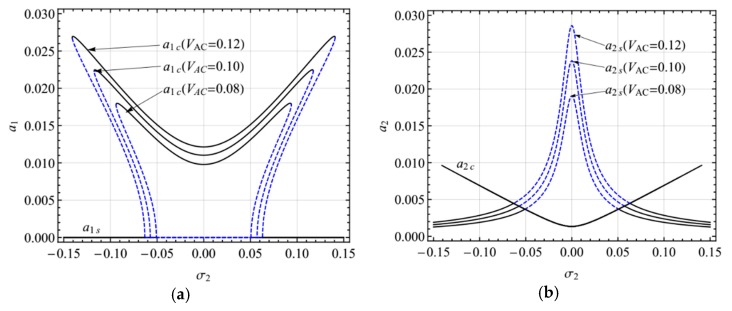
Frequency-response curves of the primary resonance of the second mode for *V*_AC_ = 0.08, 0.10, 0.12; (**a**) the first mode, (**b**) the second mode.

Figure 7 shows the frequency-response curves of the primary resonance of the second mode for different AC voltage amplitudes in the presence of two-to-one internal resonance (*V*_AC_ = 0.08, 0.10, 0.12). There are two kinds of solutions: the single-mode solution (*a*_1_ = 0, *a*_2_ ≠ 0 depicted with *a*_1*s*_, *a*_2*s*_) and coupled nonlinear solution (*a*_1_ ≠ 0, *a*_2_ ≠ 0 depicted with *a*_1*c*_, *a*_2*c*_) due to the internal resonance. In Figure 7a, the double-jumping phenomenon can be seen clearly, and the response amplitude increases with the excitation amplitude. In Figure 7b, the amplitude of the coupled nonlinear solution remains unchanged for different AC voltages, which can be regarded as the saturation phenomenon. Moreover, the single-mode solution is unstable near *σ*_2_ = 0. When the AC voltage frequency is far away from *σ*_2_ = 0, only the single-mode solution, *a*_2_, exists. 

**Figure 8:**
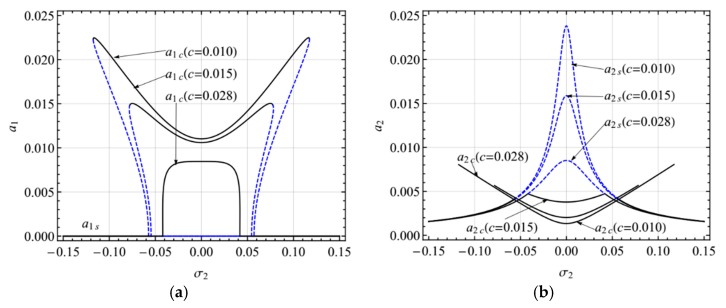
Frequency-response curves of the primary resonance of the second mode for *c* = 0.010, 0.015, 0.028; (**a**) the first mode, (**b**) the second mode.

Figure 8 shows the frequency-response curves of the primary resonance of the second mode for different damping coefficients (*c* = 0.010, 0.015, 0.028). In the first mode, as shown in Figure 8a, as the damping coefficient increases, the two peaks diminish and then reduce to one peak without jumping. Moreover, the response amplitude decreases when the damping coefficient rises. In the second mode, as shown in Figure 8b, as the damping coefficient increases, the response amplitude of the single-mode solution decreases and that of the coupled nonlinear solution decreases. 

**Figure 9:**
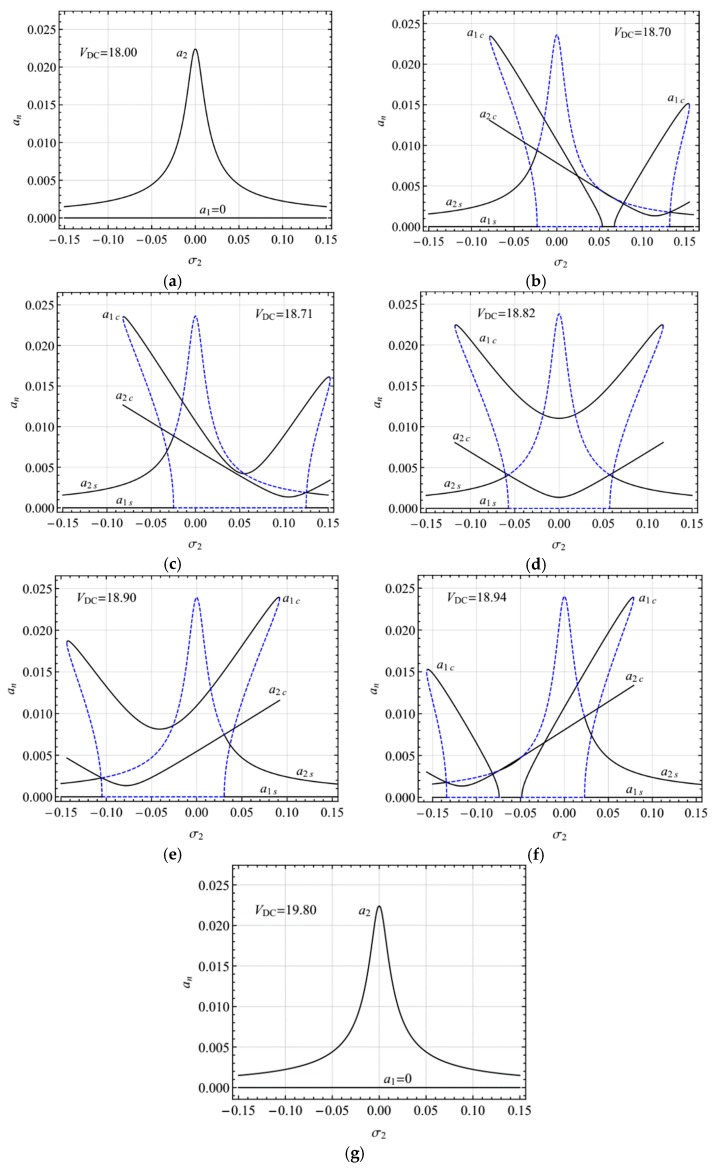
Frequency-response curves of the primary resonance of the second mode for different DC voltages: (**a**) *V*_DC_ = 18.00 (σ_1_ = -0.75), (**b**) *V*_DC_ = 18.70 (σ_1_ = -0.11), (**c**) *V*_DC_ = 18.71 (σ_1_ = -0.10), (**d**) *V*_DC_ = 18.82 (σ_1_ ≈ 0), (**e**) *V*_DC_ = 18.90 (σ_1_ = 0.08), (**f**) *V*_DC_ = 18.94 (σ_1_ = 0.11), (**g**) *V*_DC_ = 19.80 (σ_1_ = 1.00).

Figure 8 depicts the frequency-response curves of the primary resonance of the second mode for different DC voltages. For sufficiently small DC voltages, *V*_DC_, *σ*_1_ is so small that the system is far away from the two-to-one internal resonance. Hence, there is only the linear or single-mode solution (*a*_1_ = 0, *a*_2_ ≠ 0), as shown in Figure 9a. With increasing DC voltage, there are two separated jumping branches with hardening- and softening-spring types in the first mode, as shown in Figure 9b. When the DC voltage reaches a specific value, the two jumping branches merge, as shown in Figure 9c. When further increasing the DC voltage so that the system has the exact two-to-one internal resonance (*σ*_1_ = 0), the response becomes symmetrical, as shown in Figure 9d. With increasing DC voltage, so that *σ*_1_ becomes positive and much larger, the left jumping branch becomes much smaller, as shown in Figure 9e,f. With increasing DC voltage, *σ*_1_ becomes large enough so that the internal resonance condition cannot be met again and the jumping with the softening-spring type disappears, and, therefore, the response becomes single-mode (*a*_1_ = 0, *a*_2_ ≠ 0), as shown in Figure 9g.

**Figure 10:**
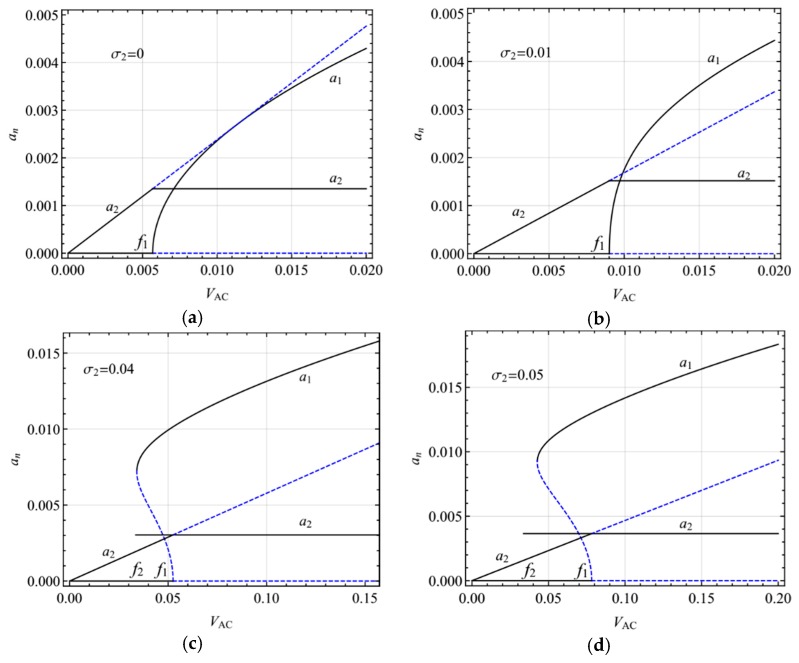
Response amplitude varying with the exciting amplitude in the primary resonance of the second mode for different exciting frequencies: (**a**) σ_2_ = 0; (**b**) σ_2_ = 0.01; (**c**) σ_2_ = 0.04; (**d**) σ_2_ = 0.05.

Figure 10 shows the variation of the amplitude of the response with the amplitude of the excitation in the presence of the two-to-one internal resonance. For the small AC voltage frequency in Figure 10a,b, when *f* < *f*_1_, there is only the single-mode solution. When *f* > *f*_1_, the amplitude of the second mode remains constant, while the amplitude of the first mode increases with the growth of excitation. In other words, there is a saturation phenomenon. For large AC voltage frequencies in Figure 10c,d, for *f* < *f*_2_, there is only the single-mode solution. For *f*_2_ < *f* < *f*_1_, the responses are either the single-mode solution or coupled two-mode solution. For *f* > *f*_1_, there exists only the coupled two mode solution. Moreover, the hysteresis phenomenon exists, as shown in Figure 10c,d.

### 4.3. Numerical Verification

**Figure 11:**
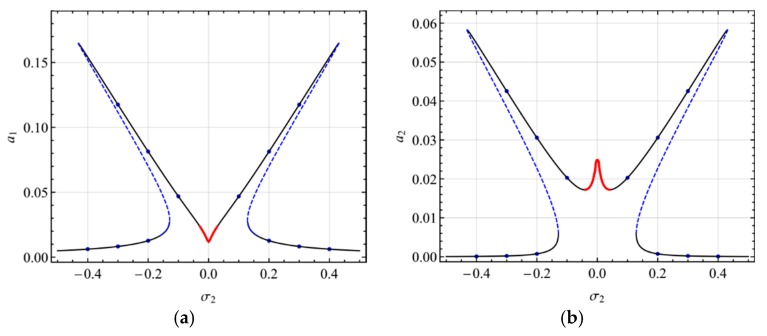
Comparison of the frequency-response curves of the primary resonance of the first mode; (**a**) the first mode, (**b**) the second mode.

**Figure 12:**
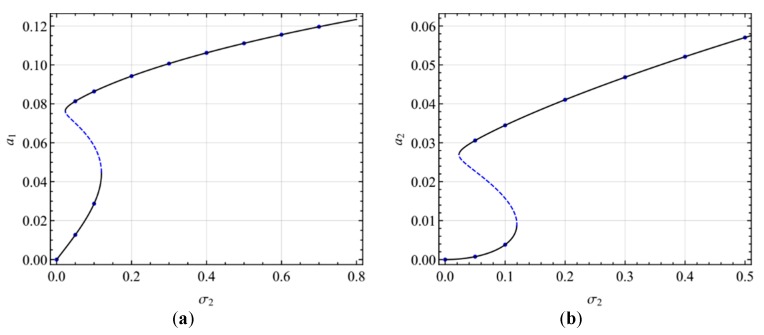
Comparison of the amplitude with forcing amplitude in the case of the primary resonance of the first mode; (**a**) the first mode, (**b**) the second mode.

**Figure 13:**
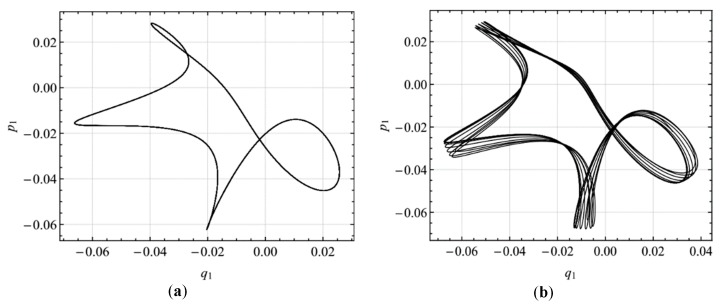
Two-dimensional projections of the phase portraits onto the *p*_1_–*q*_1_ plane when σ_2_ is between the Hopf bifurcation points; (**a**) σ_2_ = -0.0315, (**b**) σ_2_ = 0.

**Figure 14:**
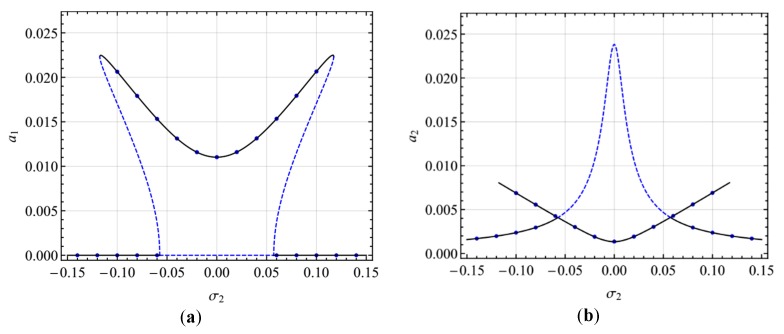
Comparison of the frequency-response curves of the primary resonance of the second mode; (**a**) the first mode, (**b**) the second mode.

**Figure 15:**
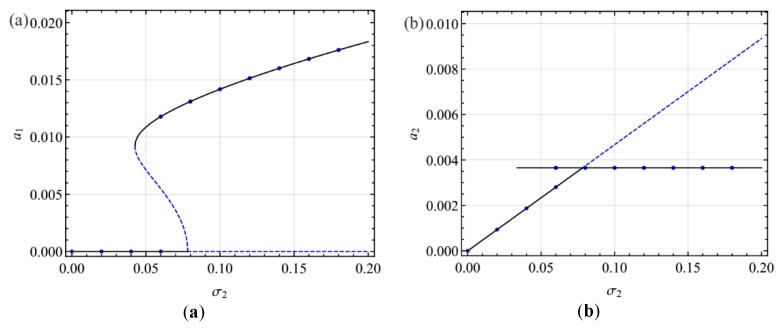
Comparison of the amplitude with forcing amplitude in the case of the primary resonance of the second mode; (**a**) the first mode, (**b**) the second mode.

To verify the steady-state solutions, we integrate numerically the modulation equations, Equations (29)–(32) of the primary resonance of the first mode and Equations (46)–(49) of the primary resonance of the second mode using the Runge-Kutta technique. Figure 11, Figure 12, Figure 13, Figure 14 and Figure 15 show the comparison of the results obtained by the multiple scales method and the numerical method. The numerical results verify the double-jumping, hysteresis, and saturation phenomena. For the amplitude of the steady-state response, the numerical and analytical results are in agreement. Moreover, to verify the modulated solution in the case of primary resonance of the first mode, Figure 13 shows the two-dimensional projections of the phase portraits onto the *p*_1_–*q*_1_ plane when σ_2_ is slightly beyond the Hopf bifurcation point, that is, σ_2_ = -0.0315 and when σ_2_ = 0. It is found that the response is modulated. This is in agreement with the results predicted by the multiple scales method.

## 5. Conclusions

The work explores the nonlinear vibration of an electrically actuated shallow arch with the flexible supports in the presence of the two-to-one internal resonance. The multiple scales method was used to solve the governing equation. The response of the system is shown by the frequency-response curves. The effect of various parameters on the response is investigated. Results showed that the first and second mode can be coupled due to two-to-one internal resonance. The double-jumping, hysteresis, and saturation phenomena can occur due to the existence of internal resonance. Moreover, the analysis predicts that by adjusting the DC voltage, the frequency-response curves transmit from single-jumping with hardening spring type to double-jumping, and further to single-jumping with softening spring type. In addition, the analytical results are supports by the numerical method.
